# Towards Miniaturization of Magnetic Gears: Torque Performance Assessment

**DOI:** 10.3390/mi9010016

**Published:** 2017-12-31

**Authors:** Efren Diez-Jimenez, Rocio Sanchez-Montero, Miriam Martinez-Muñoz

**Affiliations:** Departamento de Teoría de la Señal y Comunicaciones, Universidad de Alcalá, Ctra. Madrid-Barcelona, Km 33,66, Alcalá de Henares 28871, Spain; rocio.sanchez@uah.es (R.S.-M.); miriam.martinezm@uah.es (M.M.-M.)

**Keywords:** magnetic gears, MEMS friction, micromagnets

## Abstract

Magnetomechanical components can be a good solution in order to reduce, or even completely avoid, friction phenomena in micro-electro-mechanical systems (MEMS) since they can transmit forces through magnetic fields without contacts. In this communication, electromagnetic simulations of the expected specific torque of a coaxial magnetic gear are given. The results show that micromagnetic gears (3 mm of diameter) could provide a specific torque up to 8.98 Nm/kg, several times larger than the specific torque that microgears (<9 mm of diameter) can provide. This implies that micromagnetic gears could provide speed conversion without contact in the teeth, avoiding corresponding friction, but also that it would even improve the specific torque transmission with respect to contact microgears.

## 1. Introduction

Most micro-electro-mechanical systems (MEMS) typically contain moving elements that are susceptible of suffering from friction, wear, and fatigue. In general, for any machine, friction and wear are undesired phenomena because they limit their efficiency and reduce their lifetime. Friction and wear phenomena in microscale are primarily controlled by surface forces (such as capillary, van der Waals, electrostatic, or frictional forces) and surface properties (i.e., surface roughness and adhesion) [1]. 

For macroscale mechanical elements, friction forces are much smaller than inertia ones and therefore conventional lubrication methods are good enough for a high efficiency and long lifetime of gears. However, as long as the size is reduced, friction forces become more significant and the efficiency decreases abruptly.

Wear issues are also increased in the microscale. Material chips coming from the contact pairs and from solid lubricants. The accumulation of this debris may cause galling [2]. High levels of friction and wear are problems which currently limit the development of micro-electro-mechanical systems (MEMS) [3]. 

Many MEMS surfaces are impossible or impractical to protect from their operating environment. As a result, not only water vapor, but also other contaminants inevitably interact and stick. Friction and wear determines reliability of MEMS which is a crucial aspect [4,5,6]

The problem of wear and friction in MEMS/NEMS has been faced mostly with techniques from the surface engineering field [7]. Scale, coating, surface roughness, and lubricant influence on the friction and wear device properties. Several works have analyzed those effects [8] explaining the differences in tribological behavior from micro to nanoscale [9,10]. Although, these studies conclude with very interesting enhancements, friction and wear always appears since contact is never avoided.

Magnetomechanical components could be a good solution in order to reduce, or even completely avoid, friction phenomena in MEMS. They transmit forces using magnets and magnetic materials that exert force without contact between moving parts. Any kind of kinematic pair or mechanism is susceptible of conversion to its magneto-mechanical equivalent. Spur gears [11], planetary [12], harmonic-drives [13,14,15,16], couplings [17], bevel gear [18], cycloidal gear [19,20] can be converted to its magnetic equivalent. Not only gears can be converted but also bearings, (both rotary and linear), springs [21], suspensions, and even structures can be created with this technology.

Most of these components have been already tested demonstrating their feasibility in macroscale [22,23,24,25] avoiding friction in the teeth transmission or in the bearings. Furthermore, they can be/have been combined (bearings, gear, and coupling) as demonstrated in the first contactless machine ever built [26]. All these components are completely passive, no control or currents are involved: they behave as conventional mechanical elements but with additional advantages: no friction on teeth contacts, no lubrication is needed, wider operational temperature ranges, overload protection, and through-wall coupling connection.

All these advantages are inherent to any kind of magneto-mechanical system. Nevertheless, for the macroscale, the main drawback for their wide adoption has always been their lower specific capacity for torque/force transmission with respect to conventional ones [27]. Some macroscale developments use hybrid mechanisms, as the magnetic gears recently developed for space [28], replacing the most critical element by the magnetic equivalent and keeping other conventional elements like bearings. In this case, friction is not completely eliminated but greatly reduced while keeping a high specific torque (Nm/kg).

In the microscale, research on magneto-mechanisms are scant. Besides magnetic couplings [29] which do not really eliminate friction, there can be found only one work related to magnetomechanical components in the microscale [30]. In that work, a low specific torque spur micromagnetic gear is designed and built adequately. However, there is not any research related to coaxial magnetic gears in the microscale, although they reach the best performance at macro.

In this letter, a specific case in where a magnetomechanical component improve the performance of current micromechanical gears is explored. The design and the torque simulation of a coaxial micromagnetic gear (CMG) is provided and compared with conventional gear and microgear torque performance. 

## 2. Micromagnetic Gear Design

CMG has been selected because it has demonstrated good torque performance in the macroscale and also, because its stack topology is convenient to typical epitaxial MEMS fabrication processes. 

CMG consists of an input or fast rotary element made by several permanent magnets (PM), an intermediate element, typically static, made of soft magnetic material and a third element acting as output or slow rotary element made again of a set of PM. 

**Figure 1:**
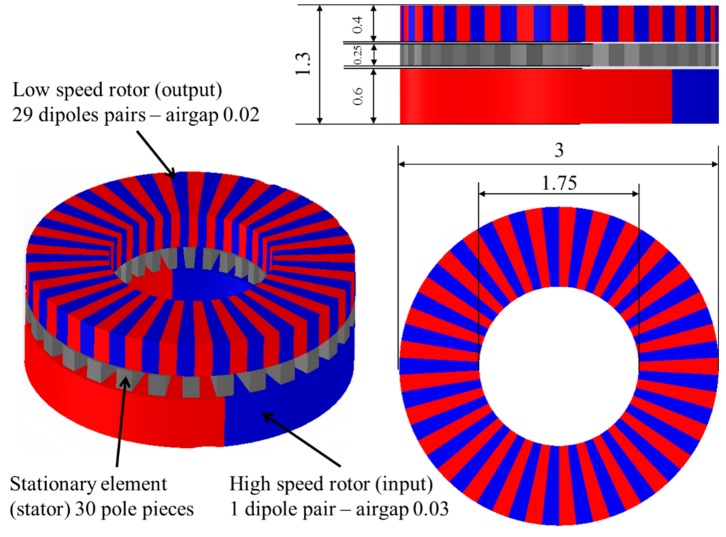
Micromagnetic gear design (dimensions in mm).

The PMs of the design shown in Figure 1 are polarized in alternative orientations indicated with the colors blue and red. All the polarizations of the PM are in the vertical direction pointing upwards (blue—north pole) or downwards (red—south pole). This type of polarization has been demonstrated feasible by previous authors [31]. However, their application to this specific design remains open research question. The number of dipole-pairs in low speed rotor is *N_output_* = 29; the number of stationary steel pole-pieces is *N_stator_* = 30; and the number of dipole-pairs in high speed rotor is *N_input_* = 1.

The gear ratio (*G_r_*) is given by the relation between the different elements according to the equation

(1) $$G_{r}=\frac{N_{output}-\;N_{stator}\;}{N_{output}}$$

This expression is valid provided that the number of dipole pairs in the input is equal to the difference between *N_output_* and *N_stator_*, i.e., *N_stator_* = 1. In this case, *Gr* = −1/29. As soft magnetic material Vacoflux 49 (Vacuumschmelze, Hanau, Germany) (*Bsat* = 2.35 T) has been considered and NdFeB as PM.

Best NdFeB magnetic properties are *Br* = 1.4 T and *Hc* = 1030 kA/m. These values can be achieved in macroscale magnets (larger than 1000 micron side) and in thin layers not thicker than 150 microns [32] even in alternative polarizations as needed. However, it has not been demonstrated such a high magnetic product for PM with 200–1000 microns thickness [33]. In this gap, only half of this magnetic product has been achieved. Thus, improving the quality of the PM in this gap also remains as an open research topic.

## 3. Simulation Results

Two simulations have been done: the first considering best NdFeB magnetic properties and the second one considering currently achievable ones. Electromagnetic FEM software (MaxFEM open source 3D, v 0.3.5, Universidade de Santiago de Compostela, Spain) has been used for the parametric simulation. This parametric simulation has been done under static assumption with output and stator element fixed and input element rotating from 0 to 180 degrees (half turn—symmetrical).

**Figure 2:**
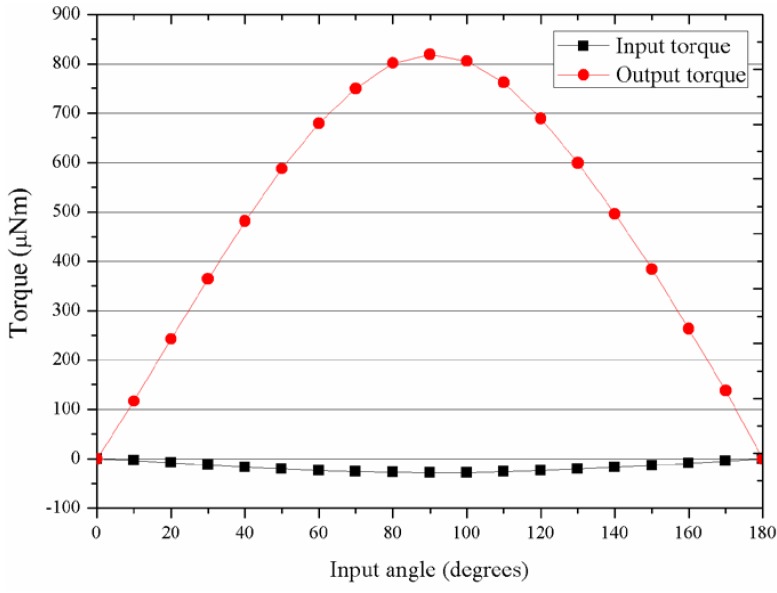
Input and transmitted torque simulation results for the best properties case.

The results, depicted in Figure 2, for the best NdFeB show a maximum peak torque in the output element of 820 μNm. The total weight of the magnetic parts is calculated in 73 mg, therefore the specific torque of the gear for a reduction ratio of −1/29 is 11.23 Nm/kg. For the case of currently achievable ones, the output element only reached 385 μNm, thus 5.27 Nm/kg.

In this preliminary design, bearings loads and design have not taken into consideration. It will certainly be necessary to add mechanical bearings, with their corresponding friction. In any case, bearings are not the most critical part in a mechanical transmission. Additionally, specific magnetic designs can be considered in order to balance bearings loads [24].

Bearings, axles, and frames will contribute with extra weight. Therefore, it is necessary to estimate final corrected specific torque values. From previous magnetic gear developments, a correction factor of 1.25 is reasonable for the final device weight [34,35]. This leads to specific torques of 8.98 and 4.21 Nm/kg.

## 4. Micromagnetic Gears vs. Conventional Gears and Microgears

The transmitted force/torque, in first term, depends directly on the amount of magnetic material bulk that the magneto-mechanism has. This implies that the specific force/torque remains constant for any size. On the contrary, for conventional mechanical elements the miniaturization increases friction/inertia force ratio, thus specific force/torque decreases when reducing size. 

**Figure 3:**
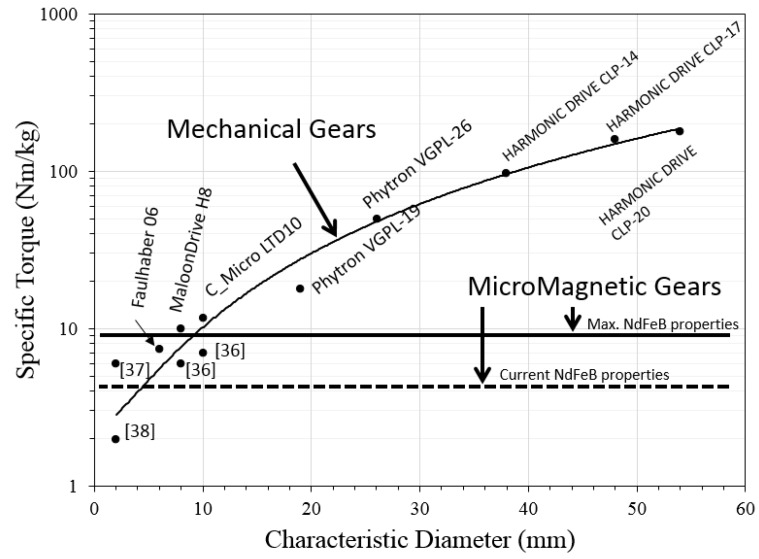
Specific torque vs. diameter of different gearsets.

Specific torques calculated for the CMG have been compared with those from different gears and microgears (Figure 3). 

The values for the comparison have been obtained from Harmonic Drive, Phytron, Faulhaber and Micromotion catalogues and also from references [36,37,38]. More than 150 commercial and research mechanical components have been scouted and considered for the comparison. Micromagnetic gears values are both for the best theoretical material properties (full line) and for the currently achievable properties (dashed line).

The tendency of the mechanical gear-specific torque values decreases quadratically with size, while those for the micromagnetic gears remain constant. This can establish a frontier wherein micromagnetic gears also would perform better in terms of specific torque. This frontier has been found around 2 mm diameter with currently achievable material properties and around 9 mm if the best material properties were achieved. 

From those dimensions, a micromagnetic gear would show better performance than a conventional microgear also in terms of mechanical torque transmission altogether with the lack of friction in the teeth meshing. This lack of friction would also allow larger operational speeds provided that, in the complete design, eddy current generation is properly mitigated. Higher specific torque transmission and their inherent advantages due to the contactless motion give micromagnetic gears enough interest to be explored for their application in MEMS. However, improvements on NdFeB magnet quality and polarization techniques are needed for the miniaturization of coaxial magnetic gears.

## 5. Conclusions

Magnetomechanical components can be a good solution in order to reduce, even completely avoid, friction phenomena in MEMS since they can transmit forces through magnetic fields without contacts. However, studies on such mechanisms are scant in the microscale.

In this letter, it has been studied the specific case of a coaxial magnetic gear. The design and the performance simulation of a micromagnetic gear are provided. The results show that micromagnetic gears could provide a specific torque up to 8.98 Nm/kg, several times larger the specific torque that previous microgears (smaller than 9 mm of diameter) can provide. 

Higher specific torque transmission and their inherent advantages due to the contactless motion give micromagnetic gears enough interest to be explored for their application in MEMS. Nevertheless, developments on micromagnet quality and polarization patterning are still needed for micro magnetic gears to become a reality. This letter tries to open or to boost some research paths that could enable novel MEMS applications using micromagnetic gears.
